# Complement inhibition ameliorates blast-induced acute lung injury in rats: Potential role of complement in intracellular HMGB1-mediated inflammation

**DOI:** 10.1371/journal.pone.0202594

**Published:** 2018-08-22

**Authors:** Yansong Li, Zhangsheng Yang, Mikulas Chavko, Bin Liu, Olawale A. Aderemi, Milomir O. Simovic, Michael A. Dubick, Leopoldo C. Cancio

**Affiliations:** 1 Department of Multiple Organ Support Technology, US Army Institute of Surgical Research, JBSA Fort Sam Houston, San Antonio, Texas, United States of America; 2 Department of Neurotrauma, Naval Medical Research Center, Silver Spring, Maryland, United States of America; 3 Department of Blood Research, US Army Institute of Surgical Research, JBSA Fort Sam Houston, San Antonio, Texas, United States of America; 4 Department of Damage Control Resuscitation, US Army Institute of Surgical Research, JBSA Fort Sam Houston, San Antonio, Texas, United States of America; Medical College of Georgia, Augusta, UNITED STATES

## Abstract

**Background and objective:**

Complement activation as an early and important inflammatory process contributes to multiple organ dysfunction after trauma. We have recently shown that complement inhibition by decay-accelerating factor (DAF) protects brain from blast-overpressure (BOP)-induced damage. This study was conducted to determine the effect of DAF on acute lung injury induced by BOP exposure and to elucidate its possible mechanisms of action.

**Methods:**

Anesthetized adult male Sprague-Daley rats were exposed to BOP (120 kPa) from a compressed air-driven shock tube. Rats were randomly assigned to three experimental groups: 1) Control (no BOP and no DAF treatment), 2) BOP (120 kPa BOP exposure), and 3) BOP followed by treatment with rhDAF (500μg/kg, i.v) at 30 minutes after blast. After a recovery period of 3, 24, or 48 hours, animals were euthanized followed by the collection of blood and tissues at each time point. Samples were subjected to the assessment of cytokines and histopathology as well as for the interaction of high-mobility-group box 1 (HMGB1) protein, NF-κB, receptor for advanced glycation end products (RAGE), C3a, and C3aR.

**Results:**

BOP exposure significantly increased in the production of systemic pro- and anti-inflammatory cytokines, and obvious pathological changes as characterized by pulmonary edema, inflammation, endothelial damage and hemorrhage in the lungs. These alterations were ameliorated by early administration of rhDAF. The rhDAF treatment not only significantly reduced the expression levels of HMGB1, RAGE, NF-κB, C3a, and C3aR, but also reversed the interaction of C3a-C3aR and nuclear translocation of HMGB1 in the lungs.

**Conclusions:**

Our findings indicate that early administration of DAF efficiently inhibits systemic and local inflammation, and mitigates blast-induced lung injury. The underlying mechanism might be attributed to its potential modulation of C3a-C3aR-HMGB1-transcriptional factor axis. Therefore, complement and/or HMGB1 may be potential therapeutic targets in amelioration of acute lung injury after blast injury.

## Introduction

Blast injury accounted for about 70% of military casualties in Iraq and Afghanistan conflicts [1,2]. Blast-induced acute lung injury (bALI) is one of several causal factors of acute respiratory distress syndrome (ARDS) in combat casualties. However, the complete understanding of the underlying molecular mechanism that regulates the development of ALI still remains obscure.

Complement activation as an early and important inflammatory process contributes to multiple organ dysfunction after trauma. Based on an array of correlative preclinical and clinical studies, it is thought that complement system activation plays a critical role in the pathogenesis of ALI [3]. Indeed, pronounced early complement activation was observed to be associated with an increased mortality rate as well with the development of ALI in patients [4]. In addition, genetic and pharmacological manipulation of complement levels and complement activation in murine models of ALI conferred beneficial effects particularly in regards to inflammation and tissue damage [5,6]. Consistent with the above findings, our previous studies have also demonstrated the beneficial effects of pharmacological manipulations of the complement activity as evident from increased survival, improved hemodynamics, reduced fluid requirements, attenuation of organ damage, and modulation of systemic and local inflammatory responses in rats and swine after trauma and hemorrhage [7–9].

As an important damage-associated molecular pattern (DAMP), extracellular High-mobility-group box 1 (HMGB1) mediates several biological consequences in inflammation, cell migration, cell proliferation and differentiation [10–12], interactions with TLR4, cytokine receptors and receptors of other signaling molecules including the receptor for advanced glycation end-products (RAGE) [13–17]. Recently, inside the nucleus or cytosol/mitochondria, additional activities of HMGB1 have been reported [18,19]. A study showed that complement activation played a crucial role in the regulation of HMGB1 release from human neutrophils [20]. Of note, the stimulation of C5L2 (a second C5a receptor) led to HMGB1 release *in vitro* and *in vivo* [21]. In line with these observations, it appears that HMGB1 might represent a pivotal molecular link between the complement cascade and inflammatory response in trauma-induced ALI.

At present the elucidation of the role of complement activation and complement inhibition on inflammation and ALI is warranted. To investigate any mechanistic link between complement machinery and inflammatory response, we assessed whether complement inhibition by recombinant human decay-accelerating factor (rhDAF) regulates inflammation and lung injury in a rat bALI model. We also explored whether the complement inhibition affects intracellular expression levels and translocation of HMGB. We hypothesized that complement inhibition by DAF mitigates blast-induced pulmonary inflammation and lung tissue injury through complement-mediated intracellular expression and translocation of HMGB1.

## Materials and methods

### Animals

Specific pathogen-free adult male Sprague-Dawley rats, weighing from 250 to 300 g were purchased from Charles River (Wilmington, MA) and used in this study and were given at least one week to acclimatize in WRAIR-NMRC vivarium. Experiments were conducted in compliance with the Animal Welfare Act at an AALAS accredited institution and in accordance with the principles of the Guide for the Care and Use of Laboratory Animals. This study was approved by the Joint WRAIR-NMRC Institutional Animal Care and Use Committee. All BOP exposure was performed under ketamine/xylazine anesthesia, and all efforts were made to minimize suffering.

### Reagents

As described previously [9], recombinant human DAF (rhDAF) and biotinylated anti-human DAF were obtained from R&D systems (Minneapolis, MN). Antibodies such as mouse anti-HMGB1, mouse anti-rat endothelial cell, rabbit anti-RAGE, Chicken anti-mouse C3/3a, and rabbit anti-NF-κB were purchased from Abcam (Cambridge, MA)., and rabbit anti-caspase-1 antibody was obtained from Cell Signaling Technologies (Danvers, MA). Mouse anti-ratC3a receptor (C3aR) was acquired from Hycult Biotech Inc (Plymouth Meeting, PA). Conjugated secondary antibodies (goat anti-mouse Alexa Fluor 488, goat anti-rabbit 594, goat anti-chicken 594), and ProLong Gold Antifade reagent were obtained from Invitrogen (Carlsbad, CA).

### Experimental design and administration of rhDAF

**Fig 1:**
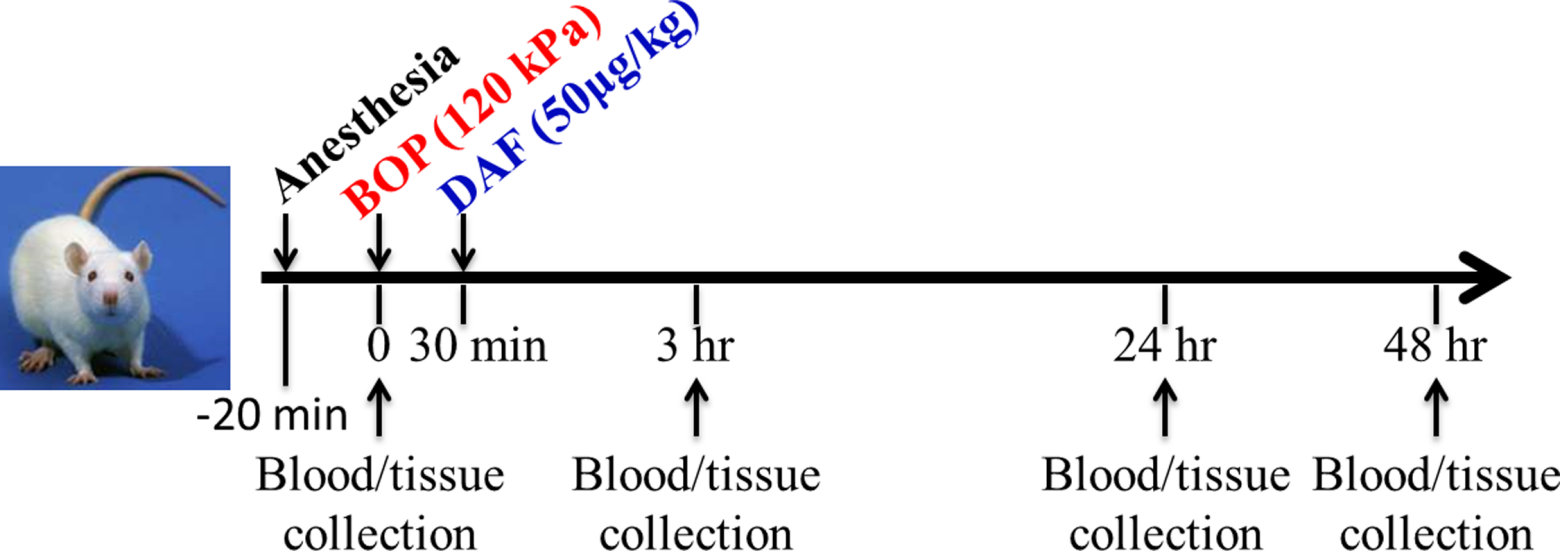
Experimental timeline. Sprague-Dawley rats were subjected to the control conditions (without injury and DAF treatment), blast exposure (BOP, BOP = 120 kPa), or blast injury treated with DAF (50μg/kg, i.v) at 30 min after BOP (designated as DAF), at indicated time points (3h, 24h, and 48h) the animals were sacrificed for blood and tissue collection.

Male rats were exposed to BOP as described previously [9,22]. Briefly, the rats were anesthetized with intra-peritoneal injection of ketamine/xylazine (60/5 mg/kg body weight) combination and placed into the end of the expansion chamber of a compressed air-driven shock tube (2.5 ft. compression chamber connected to a 15 ft. expansion chamber) and immobilized to prevent movement from blast impact and subsequent secondary or tertiary blast injuries. Animals were subjected to a single blast exposure with mean peak overpressure of 120 ± 7 kPa with their right side ipsilateral to the direction of the BOP. Animals were randomly assigned to one of three experimental groups: 1) Control, the animals underwent anesthesia, suspension, and time delays except BOP exposure (n = 8 for each time point of 3 h, 24 h and n = 5 for 48 h recovery); 2) BOP, animals were exposed to BOP followed by a bolus injection (0.5 ml of saline) via tail vein at 30 min after BOP and recovered per specified time (n = 8 for BOP-3h and BOP-24h; n = 5 for BOP-48h); 3) DAF, animals were exposed to BOP followed by a bolus injection of rhDAF (50 μg/kg body weight in 0.5 ml saline) via tail vein at 30 min after BOP and recovered at specified time point (n = 8 for DAF-3h and DAF-24h; n = 5 for DAF-48h). The experimental timeline is depicted in Fig 1. A pain assessment form was used to evaluate post-BOP pain and need for analgesic. Animals were monitored after BOP exposure by research personnel twice daily for the first 48 hours for signs of pain, distress and critical illness. At the specified time points, blood was withdrawn under ketamine/xylazine anesthesia by cardiac puncture, and serum/plasma samples were collected by centrifugation at 3,000 rpm for 10 min and stored at -80°C until used. Under general anesthesia, animals were euthanized with Fatal Plus at a dose of 150mg/kg intracardiacally and lung tissues were quickly removed and fixed with 10% formalin or 4% paraformaldehyde for histological evaluation and immunohistochemical analysis respectively.

### Histological imaging and scoring

As described previously [9], 10% neutral formalin fixed lung tissues (ipsilateral lower lobe and contralateral upper lobe) were embedded in paraffin, and sections were cut serially at a 5-μm thickness followed by staining with hematoxylin and eosin (H&E). Five random histologic images were recorded at 200× magnifications under an Olympus AX80 light microscope (Olympus, Center Valley, PA) and quantitative injury scores were graded by a pathologist blinded to the groups. The lung injury scores were calculated based on six distinct histological parameters such as pathological and morphological changes, alveolar edema, hemorrhage/congestion, inflammatory cell infiltration, alveolar septal thickening, pulmonary endothelial/epithelial damage, and thrombosis as described previously [23]. The grades for changes were assigned according to the extent (score 0, 1, 2, 3 and 4 for an extent of 0%, <25%, 25–50%, 50–75%, and 75–100% respectively) and the severity of the injury (score 0 = normal histology, score 1 = slight, score 2 = mild, score 3 = moderate and score 4 = severe alterations). The individual animal injury score was calculated for 5 images from each ipsilateral and contralateral lungs, divided by 5 for each side lung, and then averaged for each animal, and represents the sum of the extent and the severity of injury. The group injury score was averaged per total number of animals in each group.

### Immunohistochemical staining and quantification

As described previously [9], after 4% paraformaldehyde fixation, the lung tissues (ipsilateral upper/mid and contralateral lower lungs) were transferred to 20% sucrose (w/v) in PBS overnight at 4°C, followed by freezing in the Tissue-Tex OCT mounting medium (Sakura, Netherlands). Frozen tissue sections were then cut at 5-μm thickness with a cryostat and mounted onto glass slides. The tissues were fixed in cold acetone or 4% paraformaldehyde for 20 min followed by permeabilization with 0.2% Triton X-100 in PBS for further 10 min. Next, sections were blocked by 2% bovine serum albumin and incubated with primary antibodies, including anti-HMGB1, anti-RAGE, anti-NF-κB, anti-C3/3a, anti-C3aR, anti-caspas-1, and anti-DAF overnight at 4°C respectively. Following overnight incubation with primary antibodies and extensive washing, sections were incubated with secondary antibodies labeled with Alexa Fluor 488 (Green) or 594 (Red) for 1 hour at RT. Subsequently, after washing, sections were mounted with ProLong Gold Antifade solution containing 4’,6-diamidino-2-phenylindole (DAPI) for staining the nuclear DNA, and visualized under a Radiance 2100 confocal laser scanning microscope (Bio-Rad, Hercules, CA) at 200 × or 400 × magnification. Staining for negative controls was conducted by substituting the primary antibodies with corresponding immunoglobulin isotypes. Captured digital images were processed by Image J software (NIH, Bethesda, MD).

The quantification of the signals in the stained images was undertaken as previously described with minor modifications [24]. Briefly, four to six images from each animal tissue section were calibrated and adjusted using Adobe Photoshop software until only the fluorescent deposits and no visible tissue background were detected. The image was changed to black-and-white pixels with black representing the deposits of target proteins and white representing unstained areas of the image by using Image J software. Using the image Adjust Threshold command, the image was then changed to red and white, red representing the fluorescent deposits. Image analysis resulted in the red total area in pixels squared. The densitometric values for total area for all sections in each animal group were then used to determine the average area of fluorescent deposit.

### Plasma cytokine quantification

The cytokines or chemokines in the plasma from rats were measured by Bio-Plex® Pro^™^ Rat cytokine multiplex assay kit (Bio-Rad, Hercules, CA) on Luminex 200 system (Invitrogen, Carlsbad, CA) in accordance with the manufacturer’s instructions.

### Statistical analysis

All data were represented as mean ± standard error of the mean (SEM). One-way analysis of variance (ANOVA) followed by Bonferroni or unpaired *t*-test was performed by using of GraphPad Prism 5.0 (GraphPad Software, San Diego, CA). *P* value < 0.05 was considered as significant.

## Results

### rhDAF treatment significantly mitigated lung tissue injury in rats exposed to BOP

**Fig 2:**
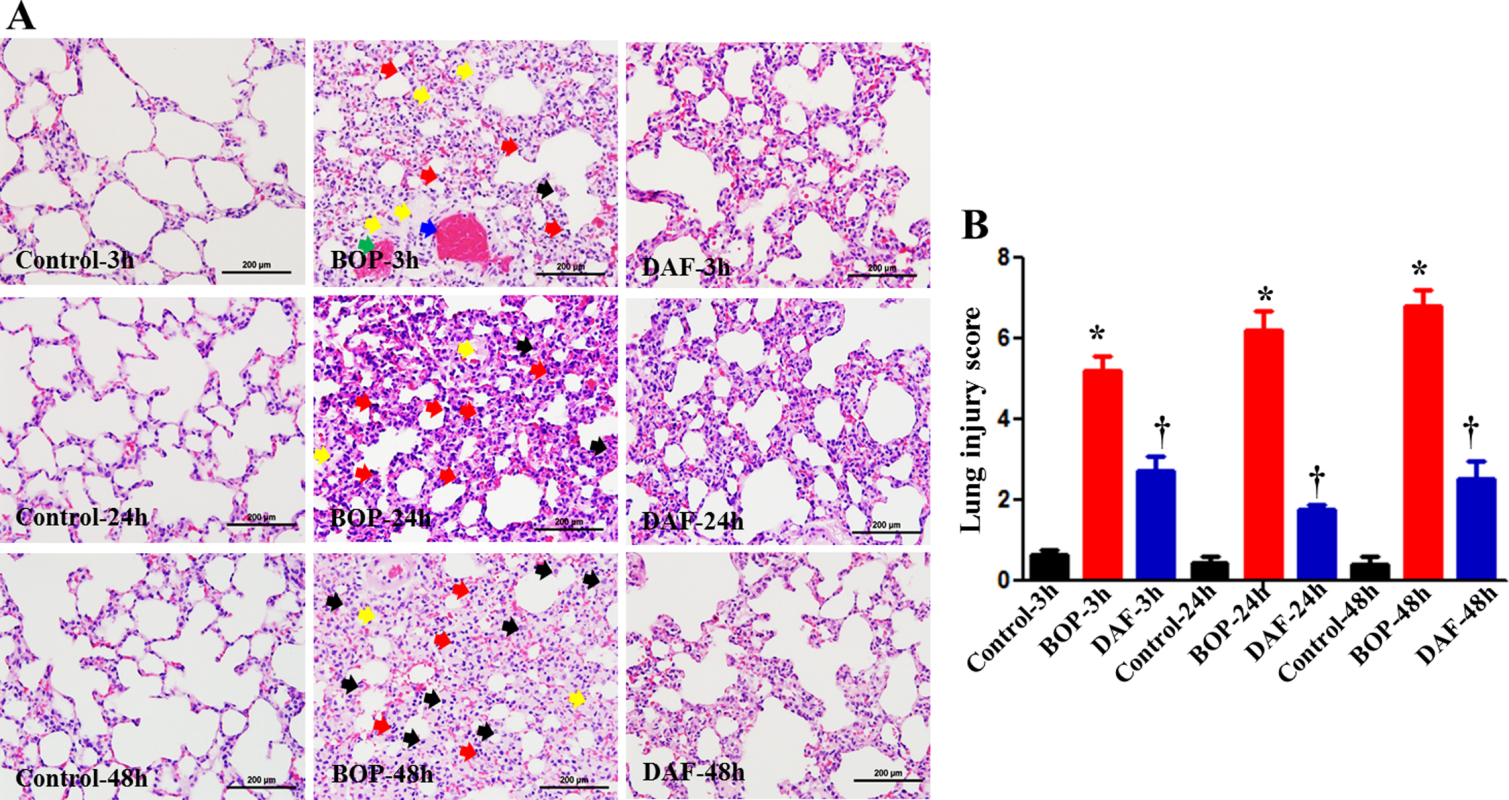
DAF treatment mitigated lung tissue injury induced by BOP in a rat model. A) Lung pathological alterations at indicated time points were evaluated in H&E-stained paraffin slide (representative images were presented, original magnification = 400×, and scale bar = 100 μm). Yellow arrows represented as alveolar edema and septal thickening, blue arrows depicted thrombus formation, green arrow represented as endothelial damage and hemorrhage, and red and purple arrows represented as neutrophils and macrophages, respectively. B) Semi-quantitative injury scores were graded based on injury criteria as described in materials and methods. The data were expressed as mean ± SEM, and one-way ANOVA followed by Bonferroni test was applied for statistical analysis of lung injury scores.

Histological analysis (Fig 2A) revealed that BOP exposure resulted in obvious alveolar septal thickening/edema (yellow arrows), inflammatory leukocyte infiltrations (black and red arrows), and endothelial damage/thrombus formation (blue arrow) and hemorrhage (green arrows), as early as 3 h after BOP, which were exacerbated at 24 h and 48 h after BOP. Specifically, a significantly more neutrophils (especially at 24 h after BOP, red arrows) and macrophages (especially at 48 h after BOP, black arrows) were observed following blast injury. rhDAF treatment, markedly attenuated these pathological injuries in the lungs. Of note, semi-quantitative pathological alteration analyses (Fig 2B) clearly demonstrated significantly lower injury scores in rhDAF-treated groups as compared to BOP injury counterpart throughout the recovery period from 3 h to 48 h.

### rhDAF treatment decreased systemic production of cytokines in rats subjected to BOP

**Fig 3:**
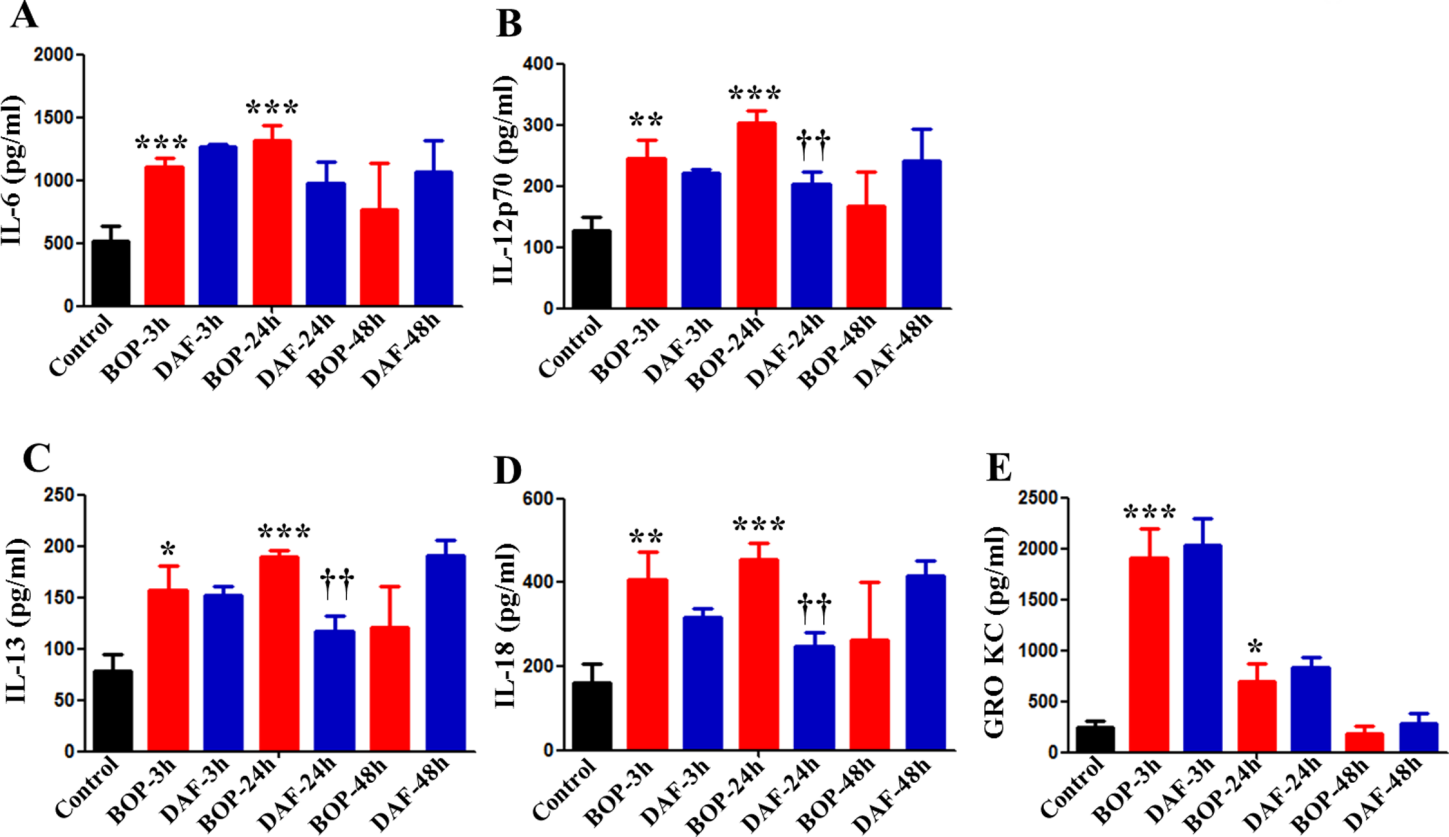
DAF treatment reduced pro-inflammatory cytokine production in rats exposed to BOP. Systemic cytokines and chemokine including IL-6 (A), IL-12p70 (B), IL-13 (C), IL-18 (D) and GRO KC/CXCL-1 (E) were measured in the blood of the animals enrolled into the experimental groups [Control, BOP, and BOP with DAF treatment (designated as DAF)] at indicated time points by Luminex® 200™ using Bio-Plex Pro™ rat cytokine multiplex assay, and group data is expressed as mean ± SEM and compared using one-way ANOVA followed by Bonferroni test. *, *p*<0.05, **, *p*<0.01, and ***, *p*<0.001 *vs*. Control. †† *p*<0.01 *vs*. BOP. n = 8 for all the groups except 48 h experimental samples (n = 5).

Since cytokines play important roles in tissue damages during blast injury, we assessed the effects of rhDAF treatment on the cytokine release by measuring the systemic cytokine and chemokine productions. We previously demonstrated that plasma levels of IL-1β, IL-10, TNF-α and EPO were increased as early as at 3 h, reaching peak at 24, and diminished at 48 h after BOP exposure, whereas DAF treatment significantly attenuated the release of IL-1β, IL-10, EPO, and RANTES, but not TNF-α, at 24 h after BOP in an animal model [9] also used in this study. In this study, multiplex assay clearly showed that levels of cytokines including IL-6, IL-12p70, IL-13 and IL-18 were significantly elevated in the plasma after blast exposure starting from 3 h and reached to peak level at 24 h followed by the decline to the baseline level at 48 h after BOP (Fig 3A–3D). The DAF treatment significantly decreased the blood levels of cytokines (IL-12p70, IL-13, IL-18) at 24 h after BOP, when compared with the BOP groups (Fig 3A–3D). The plasma level of chemokine GRO KC/CXCL-1 peaked at 3 h after BOP, but the administration of rhDAF had a little impact on plasma release of IL-6 and GRO KC (Fig 3A and 3E) in contrast to its effects on IL-12p70, IL-13, and IL-18 (Fig 3). The control-3h group was used as the control for the experimental groups at all time periods in Fig 3 since there was no significant difference in respective cytokine levels among the control groups (Control-3h, Control-24h and Control-48h) as the average cytokine values for all the three control groups were similar.

### rhDAF decreased expression and interaction of C3a and C3aR in rat lung after BOP

**Fig 4:**
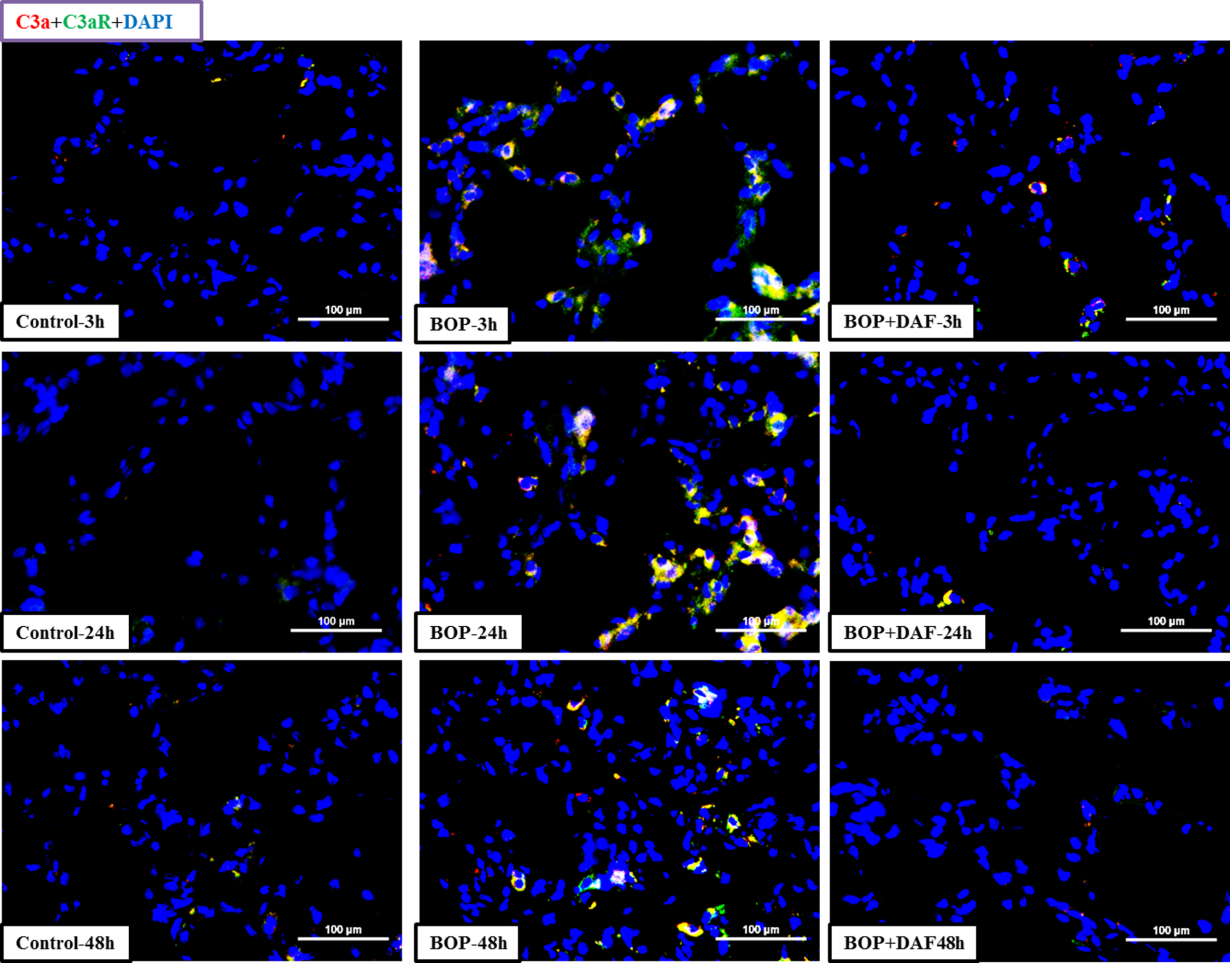
Effect of DAF on interaction of C3a and C3aR in lungs of rats after BOP exposure. Representative micro-photos of C3a-C3aR interaction in lungs of frozen sections stained with anti-C3a and anti-C3aR antibodies. Original magnification = 400×, and scale bar = 100μm. n = 8 for all the groups except 48 h experimental samples (n = 5).

Increased expression and co-localization of C3a and C3a receptor (C3aR) were observed in the lung tissue at 3, 24, and 48 h after BOP when compared to their respective controls (Fig 4). In contrast, treatment with rhDAF at 30 min after BOP led to a significant reduction in the expression and co-localization of C3a and C3aR in the lung tissue at 3 h, 24 and 48 h after blast injury (Fig 4).

### rhDAF treatment reversed the altered levels of intracellular HMGB1 and HMGB1-associated molecules in the lungs of BOP exposed rats

**Fig 5:**
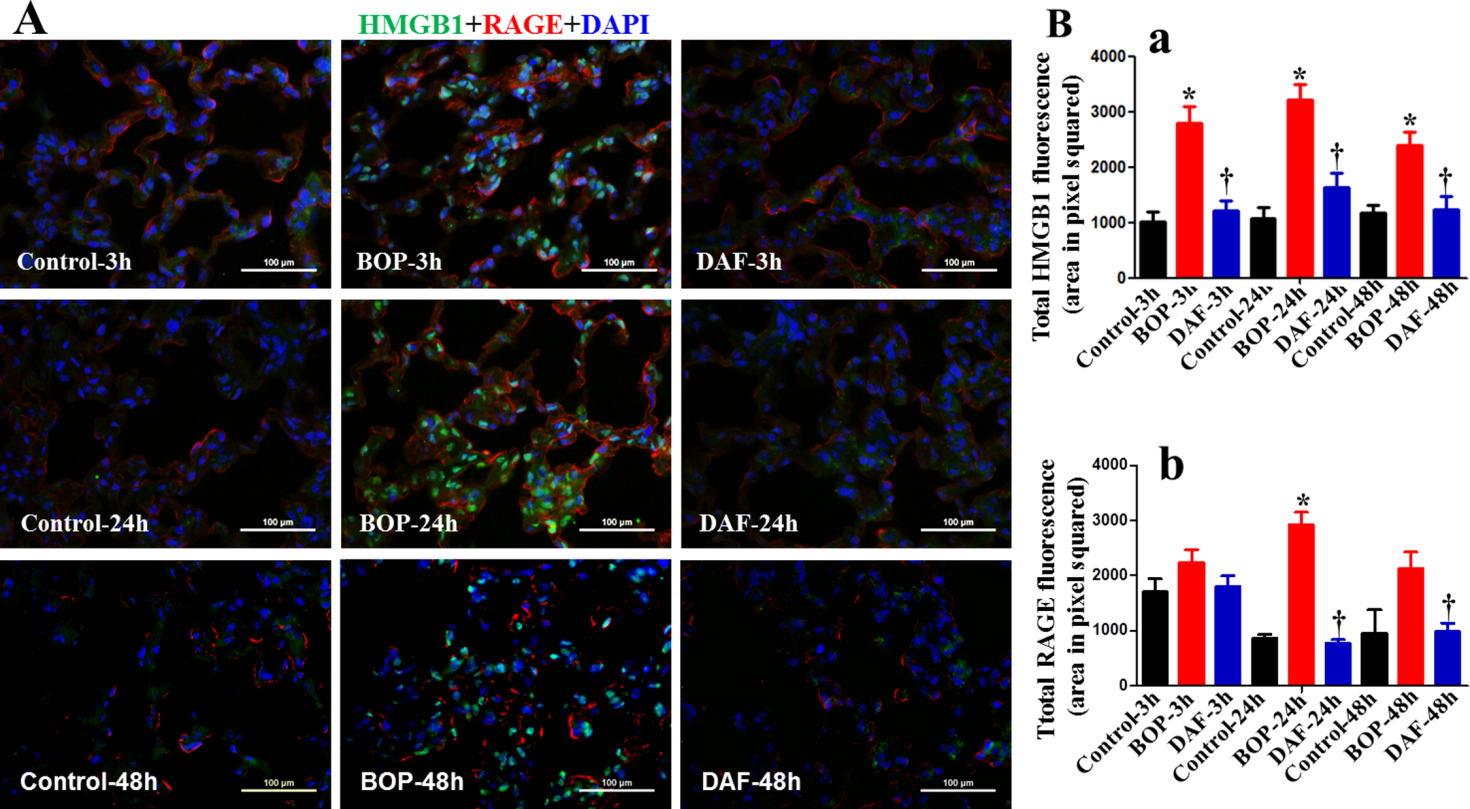
Effect of DAF on HMGB1 and RAGE expression and translocation in rats exposed to BOP. A) The expressions of HMGB1 (green) and RAGE (red) in the lung tissue were measured by Immunohistochemical staining for the groups of Control, BOP, and BOP with DAF treatment (designated as DAF) at 3 h, 24 h and 48 h after BOP and representative images were presented. Original magnification = 400×, and scale bar = 100μm. B) Mean fluorescence intensities for HMGB1 (panel a) and RAGE (panel b) from all the groups of control, BOP, and DAF at 3 h, 24 h and 48 h after BOP were quantitated with criteria as described in Materials and Methods and graphed. n = 8 for all the groups except 48 h experimental samples (n = 5). The values were expressed as mean ± SEM, and compared using one-way ANOVA followed by Bonferroni test.

As shown in Fig 5, immunofluorescent staining revealed that blast injury resulted in significantly higher expression and nuclear translocation of HMGB1 in the lung tissues at 3, 24 and 48 h after BOP (Fig 5A and 5B panel a). However, DAF treatment significantly lowered the HMGB1 expression and translocation at all the three time points (Fig 5A and 5B panel a). The HMGB1 signal can act in multiple ways to activate production of downstream inflammatory cytokines, and the HMGB1-RAGE axis plays a pivotal role in mediating inflammatory response [25]. As shown in Fig 5, BOP also increased the expression of RAGE in the lung tissue, with significantly higher levels detected at 24 h unlike at 3 and 48 h after BOP exposure (Fig 5A and 5B panel b). However, we did not observe any obvious co-localization of intracellular HMGB1 and RAGE proteins by immunofluorescence microscopy.

**Fig 6:**
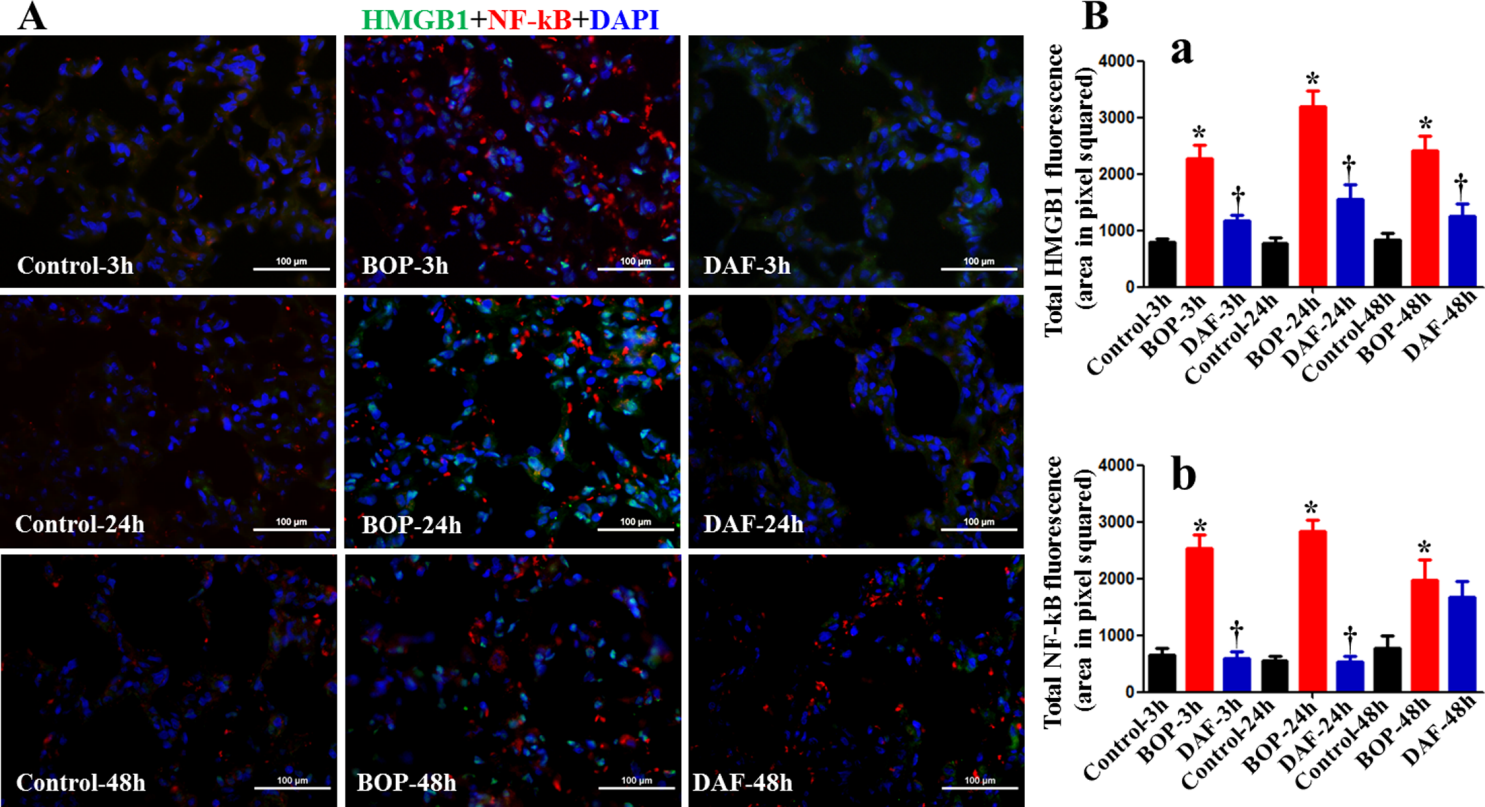
Effects of DAF on expression of HMGB1 and NF-κB in rat lungs exposed to BOP. A) The expressions of HMGB1 (green) and NF-κB (red) in the lung tissue were measured by Immunohistochemical staining for the Control, BOP, and BOP with DAF treatment (designated as DAF) at 3h and 24 h after BOP, and representative images were presented, Original magnification = 400×, and scale bar = 100μm. B) Mean fluorescence intensities for HMGB1 (panel a) and NF-κB (panel b) from all the groups of control, BOP, and BOP with DAF treatment at 3h and 24 h after BOP were quantitated with criteria as described in Materials and Methods and graphed. n = 8 for all the groups except 48 h experimental samples (n = 5). Bar graph values were expressed as mean ± SEM, and compared using one-way ANOVA followed by Bonferroni test. C) Active caspase-3 was stained with anti-active caspase-3 antibody by IHC, and mean fluorescence intensities were calculated using the criteria as described in Materials and Methods, n = 5 for each group, and the data were expressed as mean ± SEM, and compared using one-way ANOVA followed by Bonferroni test.

It is known that HMGB1 triggers inflammatory responses mainly through NF-κB activation [26]. In our studies, we also found that blast exposure induced NF-κB expression at 3, 24 and 48 h after BOP, while NF-κB expression at 3 and 24 h was lower after the DAF treatment (Fig 6A and 6B). A co-localization between HMGB1 and NF-κB was not observed, suggesting a lack of direct interaction between HMGB1 and NF-κB following blast injury in this model. There was no significant change in the expression of NF-κB at 48 h after the DAF treatment.

### Effect of rhDAF treatment on caspase-1 and active caspase-3 expression in the lungs of rats subjected to BOP

**Fig 7:**
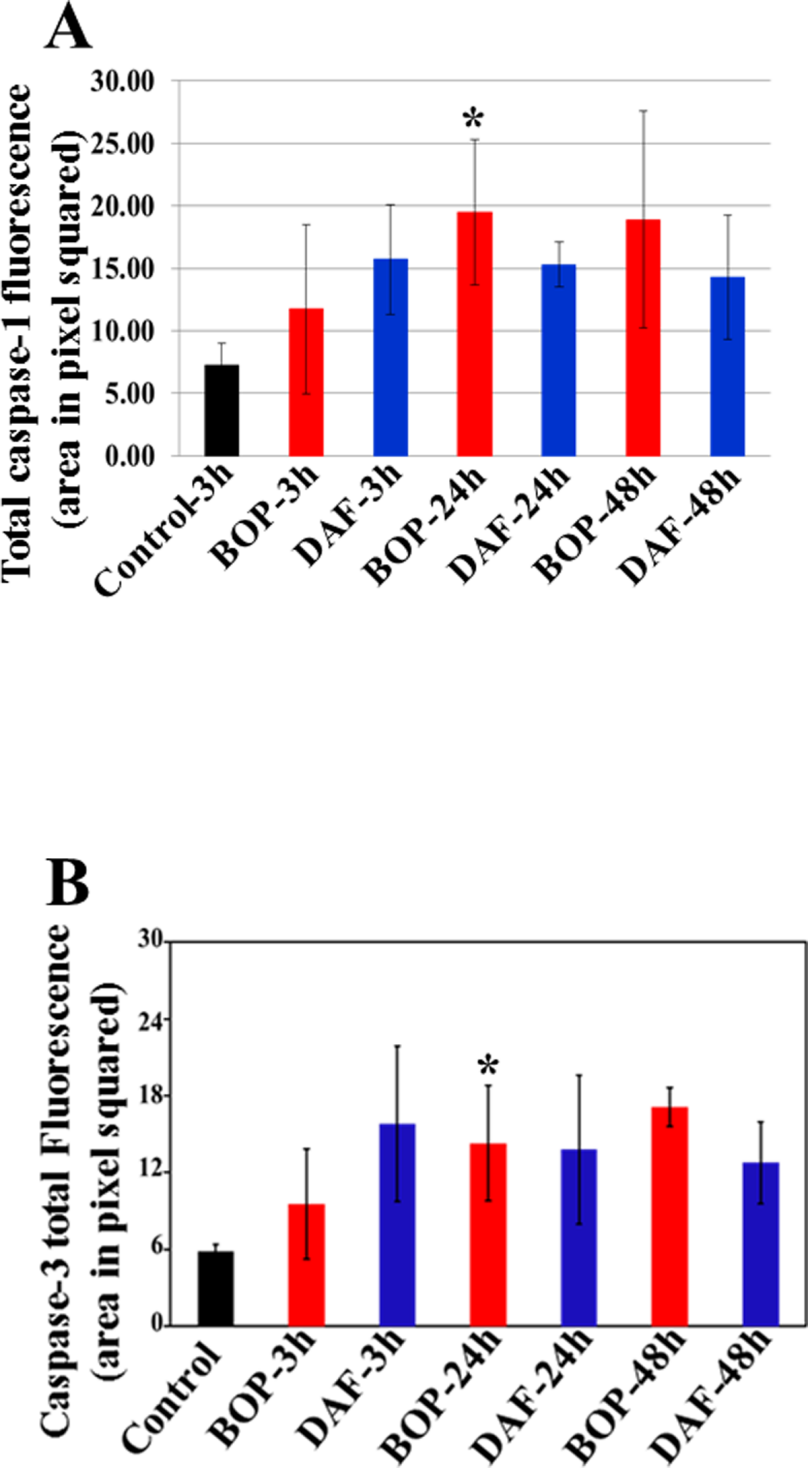
Effect of DAF on expression of caspase-3 and caspase-1 in rat lungs exposed to BOP. Caspase-1 expression was stained with anti-caspase-1 antibody by IHC, and mean fluorescence intensities were calculated using the criteria as described in Materials and Methods, n = 5 for each group, and the data were expressed as mean ± SEM, and compared using one-way ANOVA followed by Bonferroni test.

Caspase-1 plays an important role in the regulation of the generation of the key pro-inflammatory cytokines IL-1β and IL-18 in the response to cellular stress [27]. Interaction C3a-C3aR and/or C5a-C5aR have been reported to participate in caspase-1 activation in human macrophages and dendritic cells [28,29]. In this study, we measured the pulmonary expression of caspase-1. The expression of caspase-1 was significantly higher than in the control at 24 h after BOP exposure (Fig 7A). The DAF treatment trended to decrease caspase-1 levels (Fig 7A).

Given the known role of HMGB1 in regulation of apoptosis and our observation of increased HMGB1 levels after BOP, we measure cleaved caspase-3 levels to evaluate a possible link between BOP-induced HMGB1 expression and apoptosis. Our study demonstrated that active caspase-3 levels were significantly increased in the lung especially at 24 h after BOP (Fig 7B). The rhDAF treatment had no significant effect on levels of active caspase-3 level (Fig 7B). The control-3h group was used as the control for the experimental groups at all time periods (Fig 7) since there was no significant difference in the levels of caspase-1 and caspase-3 in the control groups (Control-3h, Control-24h and Control-48h), and respective mean values were similar.

### Recombinant human DAF deposits in rat lung tissue

**Fig 8:**
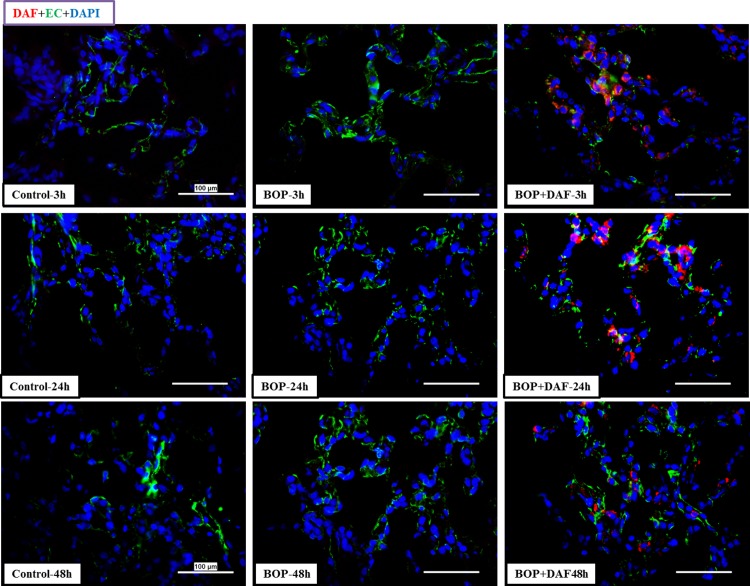
Deposition of rhDAF in rat lungs. Representative micro-photos were from frozen sections of rat lungs immunostained with anti-human DAF and anti-endothelial cell antibodies. Original magnification = 400×, and scale bar = 100μm. n = 8 for all the groups except 48 h experimental samples (n = 5).

Deposition of rhDAF in the lung was determined by immunohistochemical staining using anti-human DAF antibody. As shown in Fig 8, rhDAF deposition after administration was observed in the lungs of DAF-treated animals. Deposition of rhDAF appeared to be partially associated with the pulmonary endothelium. The rhDAF deposition was not evident in the controls and non-treated animals.

## Discussion

The principal findings of this study were as follow: 1) Early administration of DAF efficiently mitigated bALI in rats; 2) DAF significantly inhibited systemic and local inflammation; and 3) The mechanism of DAF action is probably associated with the blockage of complement-mediated intracellular expression of HMGB1 and NF-κB, and nuclear translocation of HMGB1.

Generally, blast injury is characterized by four well-documented mechanisms as follows: a) primary blast injury caused by the blast wave itself; b) secondary blast injury resulting from direct collision of impulsive and energized fragment strike; c) tertiary injury caused by blast energized directional throwing or structural collapse; and d) quaternary injury caused by flash burns/toxic gas inhalation/radiation [30,31]. Apart from these, recent studies have also demonstrated a new blast injury mechanism, termed “quinary blast injury”, which is characterized by hyper-inflammatory response, hemodynamic instability, and metabolic alterations such as bradycardia, hypotension, apnea, tissue hypoxia, oxidative stress, coagulopathy, multiple organ dysfunction, and prolonged critical illness [32]. Quinary injury may be attributed to blast-induced primary lung injury such as intrapulmonary hemorrhage and inflammation compromising pulmonary gas exchange, and thus gives rise to hypoxia, blast-triggered activation of vago-vagal reflex and neuroendocrine-immune systems. In a nutshell, the proposed pathobiology of blast-induced injury may be far more complicated than previously believed [30,31].

Blast-induced acute lung injury is still evident and a major cause of immediate death in military casualties during recent military conflicts despite the use of protective armor [33]. Unfortunately, effective therapy against bALI remains unknown because of the lack of comprehensive understanding of its pathogenesis. To gain insights on this aspect, our previous studies revealed that: 1) trauma and hemorrhagic shock initiated early complement activation and inflammatory response; 2) early complement activation was associated with multiple organ damages (lung, brain, heart, liver, kidney and intestine); and 3) early complement modulation by DAF or C1 inhibitor was able to reverse inflammatory response and corresponding cellular injury and organ damages *in vitro* and *in vivo* [7–9,23,34,35]. Specifically we discovered that DAF interrupted the C3a-C3aR axis to subsequently dampen its downstream signaling effectors such as Src kinase, caspase activity, cytokines, p-tau, and AQP-4 [9, 32]. We did not explore the intermediate signal molecules that link to the downstream signaling effectors. In the current study by employing a rat model of bBLI, we investigated 1) whether early administration of rhDAF can attenuate inflammatory responses and mitigate bALI; and 2) how DAF ameliorates inflammation and bALI.

DAF, a ubiquitously expressed intrinsic complement regulatory protein, inhibits the complement activation by preventing the assembly or accelerating the disassembly of the C3/C5 convertases in both the classic and alternative pathways thereby limiting the local C3a/C5a and C5b-9 production [9,34]. Human DAF has a structure similar to rat DAF and has displayed cross-species reactivity [36]. The selected dosage of rhDAF was in the titrated range used in the previous studies of hypoxia in rat primary neuronal cells [35], mouse ischemia-reperfusion [24,34], porcine hemorrhagic shock [8,23] and rat hemorrhagic shock (unpublished data). The time window for rhDAF administration (30 min) after blast injury used in this study was based on previous findings that systemic complement activation after a moderate BOP exposure paralleled blood-brain-barrier (BBB) breakdown as early as at 3 h, persisting up to 48 h, and returning to control levels by 72 h after the injury [37]. Intravenously administered rhDAF accumulated in the lung tissues as early as at 3 h and persisted up to 48 h following BOP (Fig 8). The distribution of rhDAF in the lungs was not mainly associated with the pulmonary endothelium, suggesting that it might directly entered into the lung tissue through damaged vasculatures and/or rhDAF-bound circulating blood cell infiltration after the blast injury.

The speculation that HMGB1 may represent a missing link primarily stems from the important role of complement in regulating inflammation. Complement C5a, a complement anaphylatoxin acted as a crucial molecule in the regulation of HMGB1 release from human neutrophils [20]. Stimulation of C5L2 (a second C5a receptor) led to the release of HMGB1 both *in vitro* and *in vivo* [21]. Clinical data demonstrated that early HMGB1 release after severe trauma in patients was associated with post-traumatic activation of complement, severe systemic inflammatory response, severity of injury, and tissue hypoperfusion [38]. Our recent findings from blast-injured combat casualties and a rat model of the combination of blast injury and hemorrhage have revealed that early HMGB1 release was correlated with early complement activation and organ injury (data not shown). On the other hand, complement protease C1s displays the ability to cleave HMGB1 into small fragments and this cleavage diminished HMGB1’s pro-inflammatory function [39]. Altogether, these data indicate an interaction between complement cascade and HMGB1, suggesting that the interaction of complement-HMGB1 might play an important role in complement-mediated inflammation and ALI after trauma.

Complement C3aR, a G-protein coupled receptor, is expressed ubiquitously, including in the lung [40,41]. The C3a-C3aR interaction is a common upstream signaling for subsequent reactions that initiates an inflammatory response. Interaction of C3a-C3aR leads to enhanced and maintained inflammatory responses such as leukocyte infiltration, vascular permeability, leukocyte activation, and inflammatory cytokine production [39]. Pathogenesis of acute lung injury has been associated with the destructive effects of C3aR-dependent signaling [42]. Consistent with our previous findings [9,35], an increased C3aR expression and interaction of C3a-C3aR were observed in lung tissue of rats exposed to BOP. Notably, C3a-C3aR engagement was markedly reduced in BOP-exposed animals treated with rhDAF, presumably through limiting local expression of C3aR and C3a and/or preventing the extravasation of systemic C3a from the pulmonary capillaries. However, the cellular and molecular signaling that underlies this pathway is unknown.

HMGB1 has multiple functions depending on the location of this protein. The HMGB1 protein is a chromosome-binding protein that was originally reported as a nuclear protein [25]. It is passively and/or actively released to the extracellular space in damaged cells as well as activated immune cells consequent to trauma, I/R injury, infection, immune disorders, neurodegenerative diseases, metabolic disorders, and cancer [25]. The extracellular HMGB1 is a key molecule of DAMPs that function as a trigger of sterile inflammation after trauma by the activation of TLR4- and RAGE-transcriptional factor pathways [12]. Recent studies showed that the activation of HMGB1-TLR4 axis and HMGB1-RAGE axis contributed to acute lung injury after trauma and hemorrhage [43,44], while blockage of extracellular HMGB1 protected inflammation-mediated acute lung injury [45]. In our study, blast injury led to increased RAGE expression on the membranes and HMGB1 expression in the intracellular compartments, but there was no direct interaction between RAGE and HMGB1. Thus, the extracellular HMGB1 appears to have little impact on BOP-triggered inflammation and ALI. However, it may not be involved in these processes. Our findings rather implicate intracellular HMGB1 in the regulation of inflammation and ALI.

Besides extracellular HMGB1 functions, intracellular HMGB1 has recently emerged as another critical molecule in regulating cellular functions. Inside the nucleus, HMGB1 functions as a DNA chaperone, nucleosome dynamics/chromosomal sustainers, and gene transcriptional modulators [18]. This posits HMGB1 to function at the intersection of upstream initiator (the complement) and downstream effector (transcription factors). In this study, we assessed the expression and translocation of HMGB1 in the lung tissue by immunohistochemistry. Our data demonstrated the nuclear translocation and accumulation of HMGB1 after BOP exposure, implying its function in the modulation of inflammatory gene transcription. In response to intracellular signaling, transcription factors such as NF-κB, a downstream component in the signaling cascade, plays a major role in regulating inflammatory responses [46]. Fan et al and Schuliga et al reported that NF-κB-regulated cytokine genes (TNFα, IL-1β, IL-2, IL-6, IL-8, IL-12, IL-13, IL-18, MCP-1, MIP-1, RANTES, GRO, etc.) are expressed in airway cells and involved in acute lung injury [47,48]. Notably, the role of C3a-C3aR- and C5a-C5aR-NF-κB axis in regulation of production and release of cytokines and chemokines has been reported [49,50]. Consistent with these reports, our previous and current data showed that the complement inhibition by DAF down-regulated BOP-induced NF-κB expression and NF-κB-regulated cytokine release (IL-1β, IL-12, IL-13, IL-18, and RANTES) [9]. These findings further indicate that NF-κB may be a key molecule in the regulation of BOP-induced inflammatory responses. Based on our previous data and this study, we postulate that complement most likely acts through the C3a-C3aR—HMGB1/NF-κB signaling cascades in regulating inflammatory response consequent to trauma. Further studies are needed to establish a comprehensively structured model.

We are aware that HMGB1 nuclear translocation following trauma appears as paradoxical with the documented data [25]. However, the prevailing notion on HMGB1 translocation suggests that its cellular localization is mainly dependent on the status of posttranslational modifications. Among the modifications, hyperacetylation on lysine residues causes HMGB1 to translocate from nucleus into the cytosol [51]. Recent studies [52] reported that trauma and hemorrhage resulted in hypoacetylation that elicits inflammatory responses through TLR4-NF-κB signaling cascade in murine models. Thus, considering the above studies, it is conceivable that the hypoacetylation status after trauma could enable HMGB1 to translocate to nucleus as we observed in our current study. Future studies are needed to examine activities of acetyl transferases and deacetylases, and HMGB1 acetylation status in our experimental model.

Diverse cellular localization of HMGB1 regarding its expression on cell membrane, cytosol, and mitochondria has been reported [25]. Mitochondria or cytosol localized HMGB1 increases autophagy, inhibits apoptosis, and regulates mitochondrial morphology and function [18,19,53]. In this study, we found that blast injury increased HMGB1 expression and nuclear translocation in the lung tiisue, whereas complement inhibition by rhDAF reversed these BOP-mediated alterations (Fig 5). Since our findings suggest the intracellular HMGB1 dynamic changes after blast injury, it is plausible that HMGB1 might also participate in intracellular homeostasis through the regulation of inflammasome, apoptosis, autophagy, and mitochondrial function. Indeed, C3a-C3aR interaction regulated IL-1β release through inflammasome activation in human monocytes at an extracellular signal-regulated kinase 1/2 –dependent fashion [28]. Our current study showed that BOP significantly increased C3a-C3aR interaction and caspase-1 expression in the lung tissue, indicating that C3a/C3aR-inflammasome axis might participate in IL-1 and IL-18 release. In our underpowered study, rhDAF treatment reduced caspase-1 expression at 24 and 48 h after BOP exposure, but the effect did not reach to statistically significant difference. Clarification of DAF on the caspase expression would require further research. Our previous *in vitro* study showed that DAF protected hypoxia-induced neuronal dysfunction and apoptosis through C3a-C3aR-Src-caspase pathway [35]. This mechanism has been further recapitulated in a rat model of blast trauma as our previous findings demonstrate that DAF administration significantly mitigates systemic and cerebral inflammation, neuronal apoptosis and degeneration, and brain injury through C3a-C3aR axis [9]. In this current study, we observed an increased expression of active caspase-3 at 24 and 48 h after BOP exposure, suggesting caspase-3 involvement and apoptosis as a late event. However, DAF had a little effect on active caspase-3 expression indicating that the complement does not have a significant role in caspase-3-mediated apoptosis. Recent study showed that cytosolic HMGB1 regulated apoptosis by protecting the autophagy protein beclin and ATG5 from calpain-mediated cleavage during inflammation [54]. It may be speculated that cytosolic HMGB1 may also participate in the modulation of apoptosis and autophagy after trauma. Another possibility that activated complement might initiate the cascade through the C3a-RAGE interaction cannot be excluded, since complement C3a can directly bind to RAGE with a high affinity [55].

## Conclusions

Our study reveals that early complement inhibition by rhDAF mitigates systemic and pulmonary inflammatory responses protecting against ALI after BOP in the rats. The underlying mechanism of DAF action is most likely orchestrated through the modulation of a signaling cascade of C3a-C3aR-HMGB1/NF-κB. A direct link between HMGB1 and NF-κB is not investigated in this research. Future studies on understanding the molecular basis and precise role of HMGB1 in these processes are required. Insight into these molecular cascades will be instrumental in discovering new therapeutic targets and developing effective treatments for trauma patients.

### Disclaimers

The opinions or assertions contained herein are the private views of the authors and are not to be construed as official or as reflecting the views of the Department of the Army or the Department of Defense.

## Supporting information

S1 Supporting Document NC3Rs ARRIVE guidelines checklist. (PDF)
